# Detecting True Change in Keratoconus after Intracorneal Ring Segment Implantation

**DOI:** 10.3390/life13040978

**Published:** 2023-04-10

**Authors:** Francisco Arnalich-Montiel, Carlota Fuente, Clara Auladell, Sara Ortiz-Toquero

**Affiliations:** 1 Department of Ophthalmology, Ramón y Cajal University Hospital, Carretera de Colmenar Viejo Km 9, 100, 28034 Madrid, Spain; 2 Optometry Research Group, IOBA-Eye Institute, Department of Theoretical Physics, Atomic and Optics, University of Valladolid, 47011 Valladolid, Spain

**Keywords:** intrastromal corneal ring segments, keratoconus, Scheimpflug camera, cutoff values, repeatability, reproducibility

## Abstract

Confirming the progression of keratoconus is of paramount relevance to providing the appropriate treatment. Real change should be considered consistent over time. It must be greater than the variability of the measurement of the device used to monitor the cornea. The present study aimed to assess the intraobserver repeatability and intersession reproducibility of a Scheimpflug camera in measuring corneal parameters in virgin keratoconus and intrastromal corneal ring segments (ICRS) implantation eyes to discriminate real change from measurement noise. Sixty keratoconus and 30 ICRS eyes were included. Corneal parameters were determined in three consecutive measurements and were repeated 2 weeks later. The precision within the same session for all parameters was better in the keratoconic eyes, with mean repeatability limits 33% narrower (range 13% to 55%) compared with ICRS eyes. Mean reproducibility limits were 16% narrower (range +48% to −45%) compared with ICRS eyes. The cutoff values to consider a real corneal shape change were lower for virgin keratoconic than for ICRS, except for the thinnest corneal thickness and Stage C (ABCD system), which were the opposite. Corneal tomography measurements in ICRS eyes showed worse accuracy than in virgin keratoconus, which should be taken into account by practitioners in patients’ follow up.

## 1. Introduction

Keratoconus is a bilateral asymmetric corneal ectasia characterized by central corneal thinning, corneal protrusion, irregular astigmatism, and low- and high-order aberrations of the eye [1]. After the diagnosis, the progression of the disease has been estimated to be somewhere between 20% and 35% [2]. Diagnosing a patient with progressive keratoconus is critical, given that it determines the next therapeutic step: corneal collagen crosslinking treatment. Although the consensus on the diagnosis is broad, current guidelines lack a clear definition of disease progression [3]. This uncertainty carries the risk of overtreating non-progressive disease or incorrectly withholding treatment from those who need treatment to arrest progression.

To document progression, consistent changes in several tomographic parameters have been proposed, including thinnest corneal thickness (TCT), maximum anterior curvature (Kmax), anterior and posterior radius of curvature (ARC, PRC), and several surface topographic indices [4,5,6,7]. What should be considered to be true change as opposed to measurement imprecision has been estimated based on repeatability and reproducibility studies on keratoconic eyes [4,5,6,7,8,9].

In the course of the disease, patients can be treated with intrastromal corneal ring segments (ICRS) to flatten the central cornea, treat irregular astigmatism, and improve the optical quality of the cornea and its aberrations [10,11]. Nevertheless, these patients also need serial tomographies to monitor progression [12]. There are no studies on repeatability and reproducibility in eyes with ICRS whatsoever.

The purposes of this study using a Pentacam (Scheimpflug imaging) were performed (1) to estimate the intra- and intersession limits of agreement of the most commonly used parameters to assess progression in patients with ICRS implantation; (2) to investigate whether these limits of the agreement show differences from those of keratoconic eyes without previous surgical treatment.

## 2. Materials and Methods

### 2.1. Patients

This prospective clinic-based observational study at the Department of Ophthalmology, University Hospital Ramón y Cajal of Madrid, Spain, divided patients into 2 groups: keratoconus without treatment and keratoconus with implanted ICRS. All patients were examined by a corneal specialist (F.A.M.). Keratoconus was diagnosed in patients who showed elevated mean central keratometry, abnormal posterior surface elevation, and corneal thinning by combining slit lamp examination, keratometry, and Scheimpflug tomography (Pentacam, Oculus Optikgeräte GmbH, Wetzlar, Germany). All patients with ICRS had a previous keratoconus diagnosis. The keratoconic and ICRS-implanted eyes included were severity-matched (ratio 2:1, respectively) based on the PRC (criteria “B” of the Belin ABCD keratoconus grading system) [7]. The most changes after ICRS implantation occurred in element A (anterior surface) of the Belin ABCD keratoconus classification, so that is why we used element B (posterior surface) to better match the eyes [13]. There was no restriction on selecting both eyes of the patient if they met the inclusion criteria because keratoconus is an asymmetric disease.

Exclusion criteria for all patients included the presence of any corneal disease except keratoconus, intraocular surgery within 6 months before the examination, corneal surgeries (except ICRS implantation in the ICRS group), and recent contact lens wearing (rigid contact lens within 4 weeks and soft contact lens within 2 weeks) or crosslinking treatment. Patients with severe scarring due to keratoconus were also excluded.

The study was approved by the hospital’s ethics committee and adhered to the tenets of the Declaration of Helsinki. Written informed consent was obtained from each participant.

### 2.2. Device

Scheimpflug images were acquired using a Pentacam (software version 1.20r 112, Oculus Optikgeräte GmbH, Weltzar, Germany). The Scheimpflug camera and a short-wavelength slit-light (blue LED at 475 nm) rotate together around the optical axis of the eye. The system rotates 180 degrees in approximately 2 s and captures 25 Scheimpflug slit images that contain 500 measurement points on the front and back corneal surfaces to draw a true elevation map.

### 2.3. Measurement Procedure

Three separate measurements of each cornea were taken with the Scheimpflug camera in each session. Measurements were performed according to the manufacturer’s guidelines in a darkened room after verification of instrument calibration. The participant placed their chin on the chin rest with their forehead pressed against the forehead strap. The measurement was performed immediately after performing a complete eye blink when the eye was aligned to the visual axis. The measurements obtained in the first session were used to assess intraobserver repeatability. The same procedure was repeated after 1 to 2 weeks by the same operator to determine the intersession reproducibility.

We investigated previous commonly used parameters to determine the progression of the ectatic disease, including flat keratometry (K1) [14] and steep (K2) [8,15] keratometry, corneal astigmatism (K2-K1), corneal thickness at the thinnest location (TCT) [16,17], and maximum keratometry value [15,18] (Kmax) at the anterior and posterior corneal surface. We also investigated other parameters, such as the anterior and posterior corneal elevation at the thinnest point, the D index from the Pentacam’s Belin Ambrosio Display (BAD-D), the anterior and posterior radius of curvature (ARC, PRC) taken from the 3.0 mm optical zone centered on the thinnest point, index of height asymmetry (IHA), index of surface variance (ISV) and the stages of A, B and C criteria of ABCD keratoconus classification system [7].

### 2.4. Statistical Analysis

The statistical analysis was performed using SPSS for Windows software (version 23.0; SPSS, Inc., Chicago, IL, USA) and Microsoft Office Excel (Microsoft Corp). To assess the normal distribution of the variables, the Kolmogorov–Smirnov test was employed (*p* > 0.05 indicating that the data were normally distributed). Descriptive results were given as the mean, standard deviation (SD), and 95% confidence interval (CI) of the mean. An independent Student’s *t*-test was used to compare corneal parameters between the keratoconus and ICRS groups for normally distributed variables and U Mann–Whitney for non-normally variables (*p* values less than 0.05 were considered statistically significant). Differences between measures in criteria A, B, and C of the ABCD classification system [13] were analyzed using the Wilcoxon test (*p* values less than 0.05 were considered statistically significant).

This study followed the definitions of intrasession repeatability and intersession reproducibility according to the British Standards Institute and the International Organization for Standardization [19] (Supplementary Table S1). To calculate the intraobserver repeatability, the following parameters were obtained from 2 repeat measurements of the first session: within-subject standard deviation (Sw) [20]; repeatability limit (r = 2.77 × Sw, which defines the difference between 2 measurements of the same volunteer for 95% of the observed pairs) [20]; and the intraclass correlation coefficient (ICC, classified as follows: less than 0.75 = poor agreement; 0.75 to less than 0.90 = moderate agreement; 0.90 or greater = high agreement) [21]. The limits of agreement (LoA) were calculated as the mean difference ±1.96 SD between measurements [21].

Intersession reproducibility was assessed on the one hand using the first measurement of each session (Method 1: first measurement of Session 1 vs. first measurement of Session 2) and, on the other hand, using the mean of 3 repeat measurements from each session (Method 2). The 95% limits of agreement were defined as the mean difference ±1.96 standard deviation between measurements performed during the 2 sessions [22]. The intersession within-subject standard deviation (SR), reproducibility limit (R = 2.77 × SR), and ICC were also calculated.

Differences in precision between the 2 groups were assessed by comparing the intraclass correlation coefficient using the Z-score distribution. The cutoff value for the progression in keratoconic and ICRS eyes was calculated by the upper limit of the 95% confidence interval of the reproducibility of 95% LoA.

## 3. Results

### 3.1. Patient Demographics

A total of 90 eyes from 64 patients were included in the study. Eyes were divided into two groups: keratoconus without treatment (60 eyes/39 patients) and an ICRS group (30 eyes/25 patients). There were 45 men and 19 women, with a mean age of 35.9 ± 12.0 (range: 18–67 years) in total keratoconus patients. Eyes in the keratoconus without treatment group and in the ICRS group were similar in relation to age (keratoconus group: 36.0 ± 12.6 years; ICRS group: 37.5 ± 13.3 years; *p* = 0.23), sex distribution (keratoconus group: 72% male; ICRS group: 68% male). Eyes between both groups were matched according to criterion B from the ABCD Keratoconus Grading System, including 12 eyes in Grade 0–2 (stage from 0 to 1.9), 22 eyes in Grade 2–3 (stage from 2 to 2.9), and 26 eyes in Grade > 3 (stage from 3 to 4) in keratoconus without treatment group; and 6, 11 and 13 eyes, respectively in the ICRS group (Supplementary Table S2).

Using the Pentacam, comparisons between both groups for all curvature, pachymetric, elevation parameters, or ABC staging showed no statistically significant differences (Table 1). Moreover, no statistically significant differences were found in ABCD classification (criteria A, B, and C) between measures taken at the same visit or between sessions (*p* ≥ 0.16).

### 3.2. Repeatability (Intrasession Agreement)

All analyzed variables were found to be repeatable with a high agreement according to the intraclass correlation coefficient (Supplementary Table S3). Intrasession agreements for all the analyzed variables in virgin keratoconus and ICRS eyes, expressed as within-subject standard deviation (Sw), repeatability limit (r), and 95% limits of agreement, are summarized in Table 2. Repeatability within the same session for all investigated parameters (except for ABCD classification) was better in the keratoconic without treatment eyes, with 33% narrower mean repeatability limits (median 31%, range 13% to 55%) compared with the ICRS eyes. The 95% LoA were also broader for the ICRS eyes. The ICC between groups was significantly higher for including flat keratometry, corneal astigmatism, posterior elevation, BAD-D Index, the anterior and posterior radius of curvature in keratoconus, ISV, and Stage A of the ABCD classification system (Supplementary Table S3).

### 3.3. Reproducibility (Intersession Agreement)

All analyzed variables were found to be reproducible with a high agreement according to the intraclass correlation coefficient (Supplementary Table S3). Intersession agreements for all the analyzed variables in virgin keratoconus and ICRS eyes expressed as SR, reproducibility limit (R), and 95% limits of agreement, are summarized in Table 2. The data are presented differently when using the difference between 1 measurement per session or the difference between the mean of 3 repeated measurements per session.

Precision between two separate sessions for all investigated parameters was better in the keratoconic without treatment eyes except for the thinnest pachymetry and Stage C, which was better in the ICRS eyes, and anterior radius of curvature and Stage A, which had similar values. Mean reproducibility limits (without ABCD keratoconus system) were 16% narrower in virgin eyes (median 18%, range 48% to −45%) compared with the ICRS eyes using 1 measurement per session, or 14% narrower (median 18%, range 43% to −32%) compared with the ICRS eyes when using the mean of 3 repeat measurements per session. Except for the thinnest pachymetry and Stage C, the 95% limits of agreement were also broader in ICRS eyes.

The ICC between groups was significantly higher for steep keratometry, D Index, the anterior radius of curvature in keratoconus without treatment, ISV, and Stage B of the ABCD classification system (Supplementary Table S3).

Reproducibility was also better in both groups and for all parameters when the mean of 3 repeated measurements instead of a single measurement was used. This led to a mean reduction in reproducibility limit of 24% (median 27%, ranging from 12% to 32%) for keratoconus and 26% ICRS (median 24%, ranging from 6% to 44%). Maximum keratometry was the parameter that improved less in both groups when using the mean of 3 repeat measurements between sessions (12% for keratoconus and 8% for ICRS).

Measurement errors appeared not dependent on mean values for maximum keratometry, steep keratometry, or corneal thickness at the thinnest location using 1 measurement or using the mean of 3 measurements for either keratoconic without treatment eyes or ICRS eyes based on Bland–Altman plots (Supplementary Figures S1–S3).

### 3.4. Cutoff Values to Consider a Corneal Shape Change

Estimates for corneal shape change based on 95% intersession limits of agreement in keratoconic eyes without treatment and ICRS are outlined in Table 3. For instance, a cutoff in maximum keratometry of 1.71 D implies with 95% confidence that an increase in maximum keratometry of more than 1.71 D in an eye with KC, taken at two time points, using 1 measurement at each time point, theoretically occurs due to measurement error and/or normal intersession variability in only 2.5% of imaging sessions; thus, it is more likely to be the result of progression.

The cutoff values to consider a real corneal shape change for virgin keratoconic eyes were lower than those for ICRS, except for corneal thickness at the thinnest location and Stage C, which were the other way around. When using the mean of 3 instead of a single measurement between sessions, Pentacam cutoffs decreased by a mean of 25% (median 24%; range 6–35%) in the keratoconus without treatment group and by a mean of 23% (median 22%; range 7–42%) in patients with ICRS. For maximum keratometry, the cutoff decrease was the lowest (6% for keratoconus and 8% for ICRS).

## 4. Discussion

Distinguishing true topographic change from measurement repeatability or reproducibility bias or noise of the device is essential for monitoring the progression of keratoconus and planning treatment with crosslinking. Unfortunately, there is no consensus on the definition of disease progression, and several parameters have been proposed but have not been properly validated [3]. In this study, the repeatability or reproducibility bias of Pentacam measurements in keratoconic eyes with ICRS and without treatment has been assessed, and cutoff values to consider a difference in tomography measurements a real sign of disease progression have been calculated.

Several randomized clinical trials on corneal collagen crosslinking effectiveness [15,18,23] define progressive keratoconus based on changes in the maximum keratometry value of the steepest axis on corneal topography >1.0-diopter (D) increase. However, these studies exclude advanced keratoconus or patients with previous corneal surgery, such as those with ICRS implantation. ICRS-implanted eyes are also susceptible to progression, especially in pediatric and young patients [12,24]. This study is the first to provide estimates of 95% limits of agreement for both repeatability and reproducibility of key corneal parameters using a Pentacam in patients with ICRS versus comparable virgin keratoconic eyes. The aim was to try to establish the measurement noise of the most used indicators of cone progression in ICRS-implanted eyes and therefore conclude whether the cutoff values for progression could be interchangeable for both groups.

A previous study had shown that although a 1D change in maximum keratometry might discriminate between progressive and non-progressive keratoconus in early and moderate keratoconus, it is not valid for advanced keratoconus (Krumeich 3 or above) with central keratometric readings of 53 D or above and minimal corneal thickness below 400 microns [8], in which a change of up to 3.5 D can be explained by the measurement noise.

Our study included all stages of keratoconus in which a valid topography could be obtained, classified using the posterior radius of curvature (criterion B of the ABCD keratoconus system), and furthermore, it analyzes the accuracy of the ABCD keratoconus classification system. In our cohort, we reported 95% limits of agreement with an upper 95% confidence interval of 1.7 for maximum keratometry in keratoconus and 1.85 in ICRS, a non-significant clinical difference. This result is somewhere between the maximum keratometry reproducibility limits found in patients with keratoconus by Epstein et al. [25] (1.51) and Hashemi et al. [26] (2.3) with similar baseline mean central keratometry, thinnest corneal thickness, and maximum keratometry value. Reproducibility in most of these studies is based on the agreement between 2 independent observers that take repeated images on the same day. Unlike these studies, we measured reproducibility as intersession variability, an agreement between 2 measurements on different days, which really is what we do when we examine the patient on different days to monitor progression. Interestingly, the differences in measurement noise between ICRS and keratoconus eyes were higher when the measurements were performed on the same day compared with measurements taken at different time points (intersession reproducibility).

However, according to the LoA, maximum keratometry and the anterior and posterior radius of curvature (and consequently stages A and B as they are related) did not differ much between the keratoconus and ICRS groups. We found clinically significant differences in steep keratometry, corneal astigmatism, corneal elevation at the thinnest point, and a BAD-D index with larger limits of agreement for ICRS. For instance, whereas a change over 1D for steep keratometry or corneal astigmatism would be a real change in keratoconus, for ICRS, this would need to be over 1.5 D. On the other hand, we found that although the limits of agreement were also clinically significant for the thinnest corneal thickness in these cases, ICRS showed narrower limits of agreement (and the same is applicable to Stage C as they are related). A real change would be over 20 microns in ICRS but only over 34 microns in keratoconus. Given that other studies have shown that using the mean of 3 measurements per session yielded a significantly lower variability between sessions and narrower limits of agreement [9]; in our study, however, it was like this for most parameters except for maximum keratometry.

On the other hand, it should be noted that this is the first time, in our knowledge, that the accuracy of the BAD-D index has been analyzed in a sample of keratoconus. This multivariate index is primarily used to distinguish between healthy corneas (≤1.54), suspected keratoconus, and definite keratoconus (≥2.38) [27]. However, it should be noted that this BAD-D index presents cutoff values for corneal shape change that can be considered high, so caution should be exercised if this index is used to evaluate progress.

There are some limitations to this study. Due to the sample size used, we did not distinguish precision according to the number, size, or thickness of the corneal ring segment, and they were all considered as a single group. For this reason, further studies would be necessary for the future to analyze the accuracy of corneal measuring equipment depending on the specific type of intrastromal ring implanted.

## 5. Conclusions

Although corneal tomography measurements in keratoconus corneas with ICRS showed worse repeatability and reproducibility results than in keratoconus without treatment, the presence of ICRS does not affect corneal topography precision. Eye care practitioners monitoring patients with keratoconus should be aware of the decrease in precision in eyes with ICRS in most corneal tomography parameters, except for corneal thickness at the thinnest point, and proposed cutoff values could help to classify a change in corneal tomography as real keratoconus progression.

## Figures and Tables

**Table 1:** Corneal measurements of Pentacam in keratoconus without treatment and ICRS groups.

Parameter	Group	Mean of 3 Measurements	*p*-Value
K1 (D)	Without treatment	45.00 ± 3.21	0.97
	ICRS	44.86 ± 3.43	
K2 (D)	Without treatment	48.63 ± 3.49	0.02
	ICRS	47.05 ± 3.54	
K2-K1 (D)	Without treatment	3.64 ± 1.98	0.12
	ICRS	2.31 ± 1.45	
Kmax at anterior surface (D)	Without treatment	53.66 ± 4.48	0.93
	ICRS	53.88 ± 5.82	
Kmax at posterior surface (D)	Without treatment	−8.56 ± 1.13	0.34
	ICRS	−8.53 ± 1.07	
TCT (µm)	Without treatment	480.42 ± 38.61	0.06
	ICRS	462.96 ± 32.97	
ELEF at thinnest point (µm)	Without treatment	17.70 ± 8.98	0.73
	ICRS	16.90 ± 11.88	
ELEB at thinnest point (µm)	Without treatment	40.56 ± 18.28	0.13
	ICRS	47.53 ± 24.52	
BAD-D	Without treatment	6.74 ± 2.90	0.14
	ICRS	7.63 ± 3.18	
PRC from 3.0 mm zone (mm)	Without treatment	5.29 ± 0.51	0.88
	ICRS	5.27 ± 0.57	
ARC from 3.0 mm zone (mm)	Without treatment	6.91 ± 0.51	0.55
	ICRS	7.11 ± 0.51	
IHA	Without treatment	31.73 ± 22.31	0.79
	ICRS	30.04 ± 20.54	
ISV	Without treatment	80.70 ± 33.63	0.85
	ICRS	80.85 ± 32.48	
Stage A	Without treatment	1.58 ± 0.70	0.66
	ICRS	1.54 ± 1.20	
Stage B	Without treatment	2.69 ± 1.26	0.71
	ICRS	2.80 ± 1.25	
Stage C	Without treatment	1.33 ± 0.74	0.06
	ICRS	1.66 ± 0.70	

ARC = anterior radius of curvature; BAD-D = Belin Ambrosio Display D index; ELEB = elevation of back surface; ELEF = elevation of front surface; ICRS = intrastromal corneal ring segments; Kmax = maximum keratometry; K1 = flat keratometry; K2 = steep keratometry; K2-K1 = corneal astigmatism; PRC = posterior radius of curvature; TCT = thinnest corneal thickness; IHA = index of height asymmetry (IHA); ISV = index of surface variance; Stage A-B-C from ABCD keratoconus system.

**Table 2:** Repeatability and reproducibility of corneal measurement in keratoconus without treatment (KC) and intrastromal corneal ring segment (ICRS) groups.

		Repeatability				Reproducibility							
		Two Measurements of Session 1				Method 1				Method 2			
Parameter	Group	Sw	r	SD of Diff	95% LoA	S_R_	R	SD of Diff	95% LoA	S_R_	R	SD of Diff	95% LoA
K1 (D)	KC	0.15	0.40	0.29	−0.58 to 0.55	0.21	0.58	0.37	−0.72 to 0.74	0.16	0.46	0.28	−0.61 to 0.50
	ICRS	0.20	0.54	0.43	−0.88 to 0.80	0.22	0.61	0.44	−0.90 to 0.84	0.17	0.47	0.36	−0.69 to 0.72
K2 (D)	KC	0.16	0.43	0.30	−0.62 to 0.55	0.18	0.49	0.33	−0.67 to 0.61	0.14	0.39	0.26	−0.54 to 0.49
	ICRS	0.26	0.71	0.50	−1.03 to 0.94	0.26	0.73	0.56	−1.17 to 1.04	0.19	0.54	0.37	−0.76 to 0.71
K2-K1 (D)	KC	0.21	0.58	0.36	−0.72 to 0.68	0.22	0.60	0.41	−0.86 to 0.73	0.16	0.45	0.29	−0.56 to 0.57
	ICRS	0.29	0.80	0.53	−0.97 to 1.11	0.30	0.82	0.61	−1.01 to 1.26	0.22	0.61	0.45	−0.82 to 0.90
Kmax-A (D)	KC	0.26	0.72	0.48	−1.04 to 0.85	0.36	1.01	0.72	−1.42 to 1.40	0.32	0.89	0.65	−1.22 to 1.31
	ICRS	0.38	1.05	0.70	−1.30 to 1.45	0.40	1.08	0.74	−1.52 to 1.37	0.37	0.99	0.68	−1.39 to 1.28
Kmax-P (D)	KC	0.08	0.22	0.16	−0.32 to 0.31	0.12	0.33	0.23	−0.44 to 0.46	0.08	0.23	0.17	−0.34 to 0.34
	ICRS	0.12	0.33	0.25	−0.52 to 0.44	0.15	0.42	0.29	−0.63 to 0.49	0.11	0.31	0.20	−0.47 to 0.34
TCT (µm)	KC	3.49	9.66	8.23	−15.19 to 17.06	5.76	15.97	13.56	−25.28 to 27.88	4.30	11.91	9.10	−17.39 to 18.29
	ICRS	5.06	14.03	8.59	−17.55 to 16.13	3.99	11.06	7.34	−15.07 to 13.71	3.30	9.01	6.03	−11.25 to 12.37
ELEF (µm)	KC	1.52	4.30	3.63	−7.68 to 6.54	1.89	5.33	3.69	−8.07 to 6.37	1.39	3.75	2.68	−5.59 to 4.92
	ICRS	2.24	6.19	4.41	−7.56 to 9.75	2.26	6.26	4.63	−7.63 to 10.53	1.42	3.94	3.13	−5.84 to 6.44
ELEB (µm)	KC	3.18	8.80	6.54	−12.51 to 13.14	3.44	9.53	6.39	−14.00 to 11.06	2.77	7.67	5.14	−10.85 to 9.19
	ICRS	5.77	15.99	11.09	−19.26 to 24.23	5.38	14.91	10.54	−20.01 to 21.30	3.00	8.30	7.44	−15.81 to 13.36
BAD-D	KC	0.30	0.82	0.58	−1.15 to 1.13	0.41	1.14	0.78	−1.66 to 1.40	0.28	0.78	0.51	−1.07 to 0.91
	ICRS	0.54	1.50	0.98	−1.72 to 2.11	0.61	1.69	1.09	−1.82 to 2.43	0.40	1.07	0.97	−2.00 to 1.80
PRC (mm)	KC	0.05	0.13	0.09	−0.19 to 0.15	0.06	0.18	0.11	−0.22 to 0.21	0.05	0.13	0.09	−0.18 to 0.17
	ICRS	0.07	0.20	0.13	−0.27 to 0.21	0.08	0.21	0.14	−0.33 to 0.23	0.05	0.13	0.09	−0.19 to 0.17
ARC (mm)	KC	0.05	0.14	0.08	−0.14 to 0.18	0.06	0.16	0.10	−0.18 to 0.21	0.04	0.11	0.07	−0.14 to 0.15
	ICRS	0.06	0.16	0.12	−0.28 to 0.20	0.06	0.16	0.11	−0.24 to 0.22	0.06	0.15	0.10	−0.20 to 0.21
IHA	KC	8.15	22.6	17.81	−34.05 to 35.75	8.85	24.52	18.11	−35.27 to 35.71	6.28	17.40	14.5	−28.43 to 28.40
	ICRS	10.26	28.42	18.87	−39.90 to 34.08	11.27	31.22	23.00	−48.57 to 41.59	9.10	25.21	16.6	−31.58 to 33.62
ISV	KC	1.33	3.69	2.78	−5.66 to 5.26	1.79	4.96	3.76	−8.28 to 6.45	1.48	4.10	2.83	−6.14 to 4.93
	ICRS	2.94	8.14	7.30	−13.17 to 15.43	3.44	9.53	8.14	−15.08 to 16.82	2.60	7.20	4.80	−9.03 to 9.77
Stage A	KC	0.10	0.27	0.19	−0.41 to 0.32	0.12	0.32	0.24	−0.50 to 0.45	0.08	0.23	0.17	−0.34 to 0.33
	ICRS	0.10	0.29	0.21	−0.35 to 0.47	0.12	0.32	0.25	−0.43 to 0.54	0.11	0.32	0.22	−0.42 to 0.44
Stage B	KC	0.08	0.21	0.17	−0.32 to 0.34	0.10	0.29	0.22	−0.44 to 0.42	0.08	0.20	0.15	−0.29 to 0.30
	ICRS	0.09	0.24	0.19	−0.40 to 0.35	0.10	0.29	0.33	−0.60 to 0.67	0.08	0.21	0.19	−0.32 to 0.41
Stage C	KC	0.09	0.25	0.17	−0.34 to 0.34	0.13	0.36	0.26	−0.52 to 0.51	0.09	0.26	0.17	−0.35 to 0.33
	ICRS	0.11	0.30	0.18	−0.35 to 0.36	0.08	0.21	0.14	−0.26 to 0.29	0.07	0.20	0.13	−0.27 to 0.25

ARC = anterior radius of curvature; r = repeatability limit; R = reproducibility limit; BAD-D = Belin Ambrosio Display D index; ELEB = elevation of back surface; ELEF = elevation of front surface; ICRS = intrastromal corneal ring segments; Kmax = maximum value of curvature; K1 = flat keratometry; K2 = steep keratometry; K2-K1 = corneal astigmatism; LoA = limits of agreement; PRC = posterior radius of curvature; SD = standard deviation; Sw = within-subject standard deviation; SR = within-subject standard deviation between sessions; TCT = thinnest corneal thickness; IHA = index of height asymmetry (IHA); ISV = index of surface variance; Stage A-B-C from ABCD keratoconus system. Reproducibility Method 1 was calculated used the first measurement of each session and Method 2 with the mean of the three measurements made in each session.

**Table 3:** Summary of cutoff values for corneal shape change expressed as upper 95% limits of agreement with upper 95% CI.

		Cutoff for Corneal Shape Change	
Parameter	Group	1 Measurement	Mean of 3 Measurements
K1 (D)	Without treatment	0.90	0.62
	ICRS	1.12	0.95
K2 (D)	Without treatment	0.76	0.60
	ICRS	1.41	0.96
K2-K1 (D)	Without treatment	0.90	0.69
	ICRS	1.65	1.18
Kmax at anterior surface (D)	Without treatment	1.71	1.60
	ICRS	1.85	1.71
Kmax at posterior surface (D)	Without treatment	0.56	0.41
	ICRS	0.67	0.47
TCT (µm)	Without treatment	33.90	22.33
	ICRS	18.45	16.26
ELEF at thinnest point (µm)	Without treatment	18.01	6.11
	ICRS	13.52	8.47
ELEB at thinnest point (µm)	Without treatment	13.90	11.47
	ICRS	28.10	18.16
BAD-D	Without treatment	1.75	1.14
	ICRS	3.13	2.42
PRC from 3.0 mm zone (mm)	Without treatment	0.28	0.21
	ICRS	0.30	0.23
ARC from 3.0 mm zone (mm)	Without treatment	0.27	0.18
	ICRS	0.28	0.26
IHA	Without treatment	43.75	34.83
	ICRS	56.44	44.35
ISV	Without treatment	8.11	6.19
	ICRS	22.08	12.86
Stage A	Without treatment	0.56	0.40
	ICRS	0.70	0.58
Stage B	Without treatment	0.51	0.36
	ICRS	0.88	0.53
Stage C	Without treatment	0.62	0.41
	ICRS	0.38	0.33

ARC = anterior radius of curvature; CI = confidence interval; D = Belin Ambrosio Display D index; ELEB = elevation of back surface; ELEF = elevation of front surface; ICRS = intrastromal corneal ring segments; Kmax = maximum value of curvature; K1 = flat keratometry; K2 = steep keratometry; K2-K1 = corneal astigmatism; LoA = limits of agreement; PRC = posterior radius of curvature; TCT = thinnest corneal thickness; IHA = index of height asymmetry (IHA); ISV = index of surface variance; Stage A-B-C from ABCD keratoconus system.

## Data Availability

The data presented in this study are available on reasonable request from the corresponding author.

## Supplementary Materials

The following supporting information can be downloaded at: https://www.mdpi.com/article/10.3390/life13040978/s1, Figure S1: Bland–Altman plots for repeatability and reproducibility 95% limits of agreement (LOA) for Kmax (maximal keratometry) in the keratoconic and ICRS groups; Figure S2: Bland–Altman plots for repeatability and reproducibility 95% limits of agreement (LOA) for K2 (steep corneal meridian) in the keratoconic and ICRS groups; Figure S3: Bland–Altman plots for repeatability and reproducibility 95% limits of agreement (LOA) for TCT (thinnest corneal thickness) in the keratoconic and ICRS groups; Table S1: Definitions of intrasession repeatability and intersession reproducibility according to the British Standards Institute and the International Organization for Standardization; Table S2: Summary of the number of eyes/patients included in each group based on the ABCD classification; Table S3: Intraclass correlation coefficient among the 3 measurements (intrasession and intersession) in the keratoconus without treatment and intrastromal corneal ring segment groups.
