# Supplementary material for: Detecting True Change in Keratoconus after Intracorneal Ring Segment Implantation
Source: Life (Basel). 2023 Apr 10;13(4):978. doi: 10.3390/life13040978 (PMC10142878; doi:10.3390/life13040978)
Supplement: Supplementary file 1 [file life-13-00978-s001.zip › supplementary material.pdf]

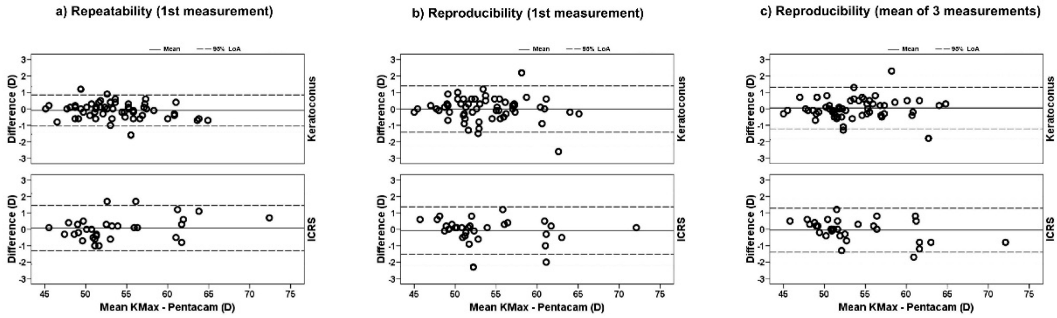

Supplementary Figure S1. Bland–Altman plots for repeatability and reproducibility 95% limits of agreement (LOA) for Kmax (maximal keratometry) in the keratoconic and ICRS groups.

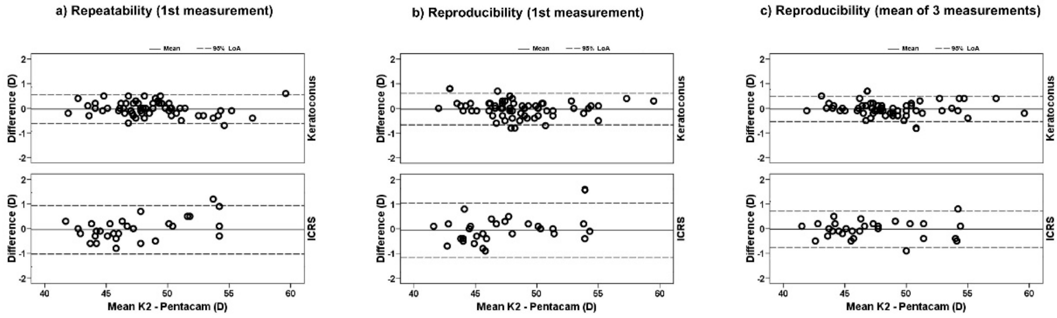

Supplementary Figure S2. Bland–Altman plots for repeatability and reproducibility 95% limits of agreement (LOA) for K2 (steep corneal meridian) in the keratoconic and ICRS groups.

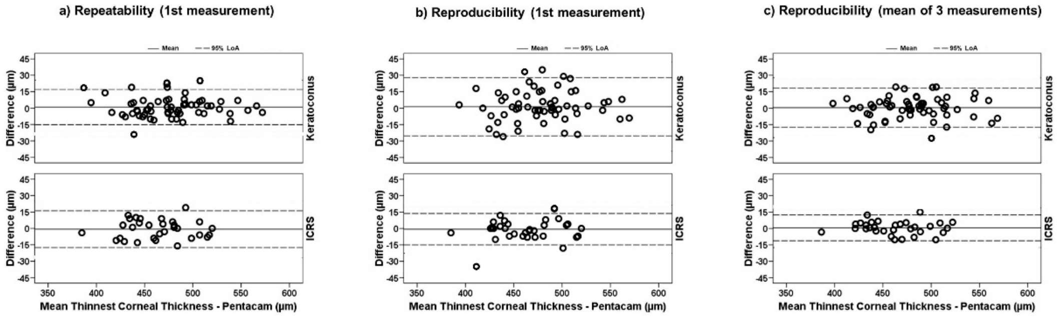

Supplementary Figure S3. Bland–Altman plots for repeatability and reproducibility 95% limits of agreement (LOA) for TCT (thinnest corneal thickness) in the keratoconic and ICRS groups.

Supplementary Table S1. Definitions of intrasession repeatability and intersession reproducibility according to the British Standards Institute and the International Organization for Standardization [20].

Parameter	Description
Sw	Intrassesion within-subject standard deviation
r	Repeatability limit (r= 2.77 × Sw, which defines the difference between 2 measurements of the same volunteer for 95% of the observed pairs)
S_R	Intersession within-subject standard deviation
R	Reproducibility limit (R= 2.77 × S_R, which defines the difference between 2 measurements of the same volunteer for 95% of the observed pairs)
ICC	Intraclass correlation coefficient (ICC, classified as follows: less than 0.75 = poor agree-ment; 0.75 to less than 0.90 = moderate agreement; 0.90 or greater = high agreement)
LoA	Limits of agreement (calculated as the mean difference ±1.96 standard deviation of the difference between measurements)

Supplementary Table S2. Summary of the number of eyes / patients included in each group based on the ABCD classification.

ABCD Classification	Group	Stage A (n eyes / n patients)	Stage B (n eyes / n patients)	Stage C (n eyes / n patients)
0	Without treatment	14 / 13	8 / 8	19 / 15
	ICRS	12 / 12	2 / 2	6 / 5
1	Without treatment	11 / 10	4 / 4	26 / 22
	ICRS	6 / 6	4 / 4	12 / 12
2	Without treatment	25 / 19	22 / 21	14 / 12
	ICRS	10 / 10	11 / 11	11 / 9
3	Without treatment	4 / 4	10 / 10	1 / 1
	ICRS	0 / 0	3 / 3	1 / 1
4	Without treatment	6 / 5	16 / 14	0 / 0
	ICRS	2 / 2	10 / 8	0 / 0

Supplementary Table S3. Intraclass correlation coefficient among the 3 measurements (intrasession and intersession) in the keratoconus and intrastromal corneal ring segment groups. ARC = anterior radius of curvature; D = Belin Ambrosio Display D index; ELEB = elevation of back surface; ELEF = elevation of front surface; ICRS = intrastromal corneal ring segments; Kmax = maximum keratometry; K1=flat keratometry; K2=steep keratometry; K2-K1=corneal astigmatism; PRC = posterior radius of curvature; TCT = thinnest corneal thickness. IHA= index of height asymmetry (IHA); ISV= index of surface variance; Stage A-B-C from ABCD keratoconus system. *Statistically significant differences in the ICC between the keratoconus without treatment and ICRS groups ($Z \geq 1.645$ is considered statistically significant).

Parameter	Group	Z	ICC Intrasession (95% CI)	Z	ICC Intersession (95% CI)
**K1 (D)**	Without treatment	1.75*	0.999 (0.998 – 0.999)	1.38	0.998 (0.997 – 0.999)
	ICRS		0.997 (0.995 – 0.998)		0.997 (0.995 – 0.998)
**K2 (D)**	Without treatment	1.23	0.999 (0.998 – 0.999)	1.75*	0.999 (0.997 – 0.999)
	ICRS		0.997 (0.994 – 0.998)		0.997 (0.994 – 0.998)
**K2-K1 (D)**	Without treatment	2.16*	0.995 (0.993 – 0.997)	2.41*	0.995 (0.991 – 0.997)
	ICRS		0.988 (0.975 – 0.994)		0.984 (0.972 – 0.992)
**Kmax at anterior surface(D)**	Without treatment	1.40	0.998 (0.997 – 0.999)	1.23	0.995 (0.992 – 0.997)
	ICRS		0.997 (0.995 – 0.999)		0.997 (0.993 – 0.998)
**Kmax at posterior surface(D)**	Without treatment	1.23	0.996 (0.994 – 0.997)	1.08	0.995 (0.993 – 0.997)
	ICRS		0.992 (0.986 – 0.996)		0.991 (0.984 – 0.995)
**TCT (µm)**	Without treatment	1.35	0.993 (0.989 – 0.995)	1.09	0.986 (0.976 – 0.992)
	ICRS		0.988 (0.978 – 0.994)		0.992 (0.983 – 0.996)
**ELEF at thinnest point (µm)**	Without treatment	0.01	0.979 (0.968 – 0.987)	0.10	0.979 (0.964 – 0.987)
	ICRS		0.979 (0.962 – 0.989)		0.978 (0.960 – 0.988)
**ELEB at thinnest point (µm)**	Without treatment	3.51*	0.986 (0.978 – 0.991)	0.33	0.980 (0.967 – 0.987)
	ICRS		0.888 (0.797 – 0.942)		0.977 (0.952 – 0.989)
**BAD-D**	Without treatment	3.72*	0.994 (0.991 – 0.996)	2.41*	0.993 (0.988 – 0.996)
	ICRS		0.922 (0.857 – 0.960)		0.977 (0.952 – 0.989)
**PRC from 3.0 mm zone (mm)**	Without treatment	3.28*	0.986 (0.979 – 0.991)	0.42	0.981 (0.974 – 0.984)
	ICRS		0.918 (0.850 – 0.958)		0.979 (0.966 – 0.985)
**ARC from 3.0 mm zone (mm)**	Without treatment	3.38*	0.993 (0.990 – 0.996)	2.65*	0.992 (0.987 – 0.995)
	ICRS		0.953 (0.914 – 0.976)		0.968 (0.933 – 0.985)
**IHA**	Without treatment	0.87	0.908 (0.858 – 0.943)	0.24	0.838 (0.748 – 0.899)
	ICRS		0.849 (0.726 – 0.922)		0.817 (0.668 – 0.906)
**ISV**	Without treatment	1.92*	0.999 (0.998 – 0.999)	1.90*	0.999 (0.998 – 0.999)
	ICRS		0.994 (0.989 – 0.997)		0.995 (0.991 – 0.997)
**Stage A**	Without treatment	1.72*	0.996 (0.993 – 0.997)	0.85	0.995 (0.993 – 0.997)
	ICRS		0.980 (0.962 – 0.990)		0.978 (0.960 – 0.988)
**Stage B**	Without treatment	1.54	0.995 (0.993 – 0.997)	1.93*	0.994 (0.991 – 0.996)
	ICRS		0.987 (0.977 – 0.993)		0.977 (0.960 – 0.988)
**Stage C**	Without treatment	0.95	0.992 (0.987 – 0.995)	0.17	0.988 (0.981 – 0.992)
	ICRS		0.987 (0.978 – 0.993)		0.987 (0.977 – 0.993)
